# Discovery of a New *Lichtheimia* (*Lichtheimiaceae*, *Mucorales*) from Invertebrate Niche and Its Phylogenetic Status and Physiological Characteristics

**DOI:** 10.3390/jof9030317

**Published:** 2023-03-03

**Authors:** Thuong T. T. Nguyen, André Luiz Cabral Monteiro de Azevedo Santiago, Paul M. Kirk, Hyang Burm Lee

**Affiliations:** 1 Environmental Microbiology Laboratory, Department of Agricultural Biological Chemistry, College of Agriculture and Life Sciences, Chonnam National University, Gwangju 61186, Republic of Korea; 2 Departamento de Micologia, Universidade Federal de Pernambuco, Av. da Engenharia, s/n, Recife 50740-4600, PE, Brazil; 3 Biodiversity Informatics and Spatial Analysis, Jodrell Laboratory, Royal Botanic Gardens Kew, Surrey TW9 3DS, UK

**Keywords:** ITS rDNA, LSU rDNA, *Lichtheimiaceae*, morphology, *Mucoromycota*, taxonomy

## Abstract

Species of *Lichtheimia* are important opportunistic fungal pathogens in the order *Mucorales* that are isolated from various sources such as soil, indoor air, food products, feces, and decaying vegetables. In recent years, species of *Lichtheimia* have become an emerging causative agent of invasive mucormycosis. In Europe and USA, *Lichtheimia* are the second and third most common causal fungus of mucormycosis, respectively. Thus, the aim of this study was to survey the diversity of species of *Lichtheimia* hidden in poorly studied hosts, such as invertebrates, in Korea. Eight *Lichtheimia* strains were isolated from invertebrate samples. Based on morphology, physiology, and phylogenetic analyses of ITS and LSU rDNA sequence data, the strains were identified as *L. hyalospora*, *L. ornata*, *L. ramosa*, and a novel species, *L. koreana* sp. nov. *Lichtheimia koreana* is characterized by a variable columellae, sporangiophores arising solitarily or up to three at one place from stolons, and slow growth on MEA and PDA at all temperatures tested. The new species grows best at 30 and 35 °C and has a maximum growth temperature of 40 °C. Detailed descriptions, illustrations, and a phylogenetic tree are provided.

## 1. Introduction

*Mucorales*, the largest order of *Mucoromycota*, includes 14 families, 55 genera, and approximately 300 described species [1,2,3]. Of these species, 38 belonging to 12 genera—specifically *Actinomucor*, *Apophysomyces*, *Cokeromyces*, *Cunninghamella*, *Lichtheimia*, *Mycotypha*, *Mucor*, *Rhizomucor*, *Rhizopus*, *Saksenaea*, *Syncephalastrum*, and *Thamnostylum*—have been reported to be involved in human infections of mucormycosis [4,5]. Members of *Rhizopus*, *Mucor* and *Lichtheimia* are the most common genera that cause this, representing 70–80% of all cases, whereas *Cunninghamella*, *Apophysomyces*, *Saksenaea*, *Rhizomucor*, *Cokeromyces*, *Actinomucor* and *Syncephalastrum* are rarely reported [6].

The genus *Lichtheimia* (*Mucorales*, *Lichtheimiaceae*) consists of saprotrophic fungi inhabiting soil, plants, indoor air, food products, and feces [1,7,8] and contains important causative agents of mucormycoses in humans and animals [7,9]. Species of *Lichtheimia* are broadly distributed in all continents, with species being isolated from environmental and clinical sources [6,9,10].

For a long time, *Lichtheimia* has been treated as a synonym for *Absidia* based on morphological similarities [11]. Hoffmann et al. [12] revised *Absidia* based on phylogenetic, physiological, and morphological characteristics and divided its constituent species into three groups: thermotolerant—optimum growth temperatures above 37 °C with a range of 37–45 °C; mesophilic—optimum growth temperatures between 25–34 °C; mycoparasitic—optimal growth temperatures below 30 °C. Based on these data, the thermotolerant species were reclassified into the genus *Mycocladus*, as follows: *Mycocladus corymbifer* (formerly *A. corymbifera*), *M. blakesleeanus*, and *M. hyalosporus*. Subsequently, these three thermotolerant species were placed in the genus *Lichtheimia* as *L. corymbifera*, *L. blakesleeana*, and *L. hyalospora* [13], with the genus typified by *L. corymbifera*. Alastruey-Izquierdo et al. [7] transferred *A. ornata* to *Lichtheimia* as *L. ornata*, described a new species, *L. sphaerocystis*, and reduced *L. blakesleeana* to a synonym of *L*. *hyalospora*. In 2014, a novel species, *L. brasiliensis*, was discovered in Brazil [8].

Currently, the genus contains six species, *L*. *corymbifera*, *L. ramosa*, *L*. *ornata*, *L*. *hyalospora*, *L*. *sphaerocystis*, and *L*. *brasiliensis* [14]. Only *L*. *corymbifera*, *L*. *ramosa*, and *L*. *ornata* have been found to be clinically relevant [15].

Several studies have explored the ability of *Lichtheimia* species to produce potential bioactive compounds [16,17,18]. For example, *L. ramosa* is known to produce different types of enzymes including xylanase, *β*-glucosidase, amylases, hemi-cellulases, and carboxy-methyl-cellulase (CMCase) [19,20,21,22,23,24]. It also produces the potential volatile metabolites such as acetic acid, ethanol, 3-methyl-2-buten-1-ol, 2-phenylethanol, ethylacetate, 2-furaldehyde, 5-(hydroxymethyl)-2-furaldehyde, 2,3-dihydro-3,5,-dihydroxy-6-methyl-4H-pyran-4-one, and α-humulene [18]. *Lichtheimia hyalospora* has been investigated for the production of chitosan and polyunsaturated fatty acids (PUFAs) [25,26].

The purpose of this study was to expand the present knowledge of fungal diversity within the order *Mucorales,* hidden in poorly studied hosts, such as invertebrates. A novel species of *Lichtheimia* is proposed based on morphological and physiological features, as well as molecular data of ITS and LSU rDNA sequences.

## 2. Materials and Methods

### 2.1. Sampling and Isolation

Invertebrate samples were collected from Kunryang-ri, Cheongyang, Chungnam Province, Korea in 2020 and 2022. The samples were collected in polyethylene containers and stored at ambient temperature during transport to the laboratory, where isolation of fungi was conducted as previously described [27,28]. Holotype and ex-type living cultures were deposited at the Environmental Microbiology Laboratory, Chonnam National University in Gwangju, Korea.

### 2.2. Morphological Studies

Pure cultures were grown in triplicate on potato dextrose agar (PDA), malt extract agar (MEA), and synthetic mucor agar (SMA) [29,30]. Microscopic characters from the isolates were examined and measured after 4 to 7 days of growth on MEA, PDA, and SMA at 25 °C and mounted in lactic acid (60%) and observed under a differential interference contrast microscope (Olympus BX53, Tokyo, Japan).

### 2.3. Growth Experiments

Strains of CNUC ISS71, CNUFC S724, CNUFC CY2204, CNUFC CY2246, CNUFC CY2248, CNUFC S871, CNUFC CY2232 and CNUFC CY2219 were grown in triplicate on SMA, PDA and MEA and incubated at 20, 25, 30, 35, 40, 41, 42, 43, 45, 46, 47, 48 and 50 °C in the dark. Colony growth was measured every 24 h and was monitored for 3 days. The maximum growth temperature (Tmax) was determined at temperatures one or two degrees higher than the last temperature with growth.

### 2.4. Mating Experiments

Mating experiments were carried out on MEA, PDA, and SMA plates at 20, 25, and 30 °C, as described by Santiago et al. [8]. Briefly, a disk about 5 mm in diameter was cut from each partner of the mating pair and placed on opposite sides of a plate. The plates were checked for zygospores for up to two months using a stereomicroscope (Leica S9i).

### 2.5. DNA Extraction, PCR, and Sequencing

Fungal isolates were cultured on PDA overlaid with cellophane at 25 °C for 4 days. Mycelia were collected by scraping the surface of the cellophane and placing this sample in sterile 1.5 mL Eppendorf tubes. Genomic DNA was then extracted using the SolgTM Genomic DNA Preparation Kit (Solgent Co. Ltd., Daejeon, Republic of Korea) according to the manufacturer’s protocol, and subsequently stored at −20 °C. Two genomic regions were amplified by PCR: the internal transcribed spacer (ITS) region was amplified using primers V9G/ITS4 and V9G/LS266 [31,32,33], and the large subunit rDNA region was amplified using primers LR0R and LR5 [34]. The reactions and conditions for PCR were as previously described [27]. The amplified fragments were purified using an Accuprep PCR Purification Kit (Bioneer Corp., Daejeon, Republic of Korea). Amplicons were sequenced in both directions with a 3730XL DNA analyzer (Applied Biosystems, Foster City, CA, USA) at Macrogen (Daejeon, Republic of Korea). The SeqMan v. 7.0 program was used to assemble and edit the raw sequences.

### 2.6. Phylogenetic Analyses

Sequences of each locus were aligned using MAFFT v. 7 with the L-INS-I algorithm (http://mafft.cbrc.jp/alignment/server, accessed on 2 January 2023) [35], then confirmed manually in MEGA v. 7 [36]. Bayesian inference (BI) and maximum likelihood (ML) analyses were performed for the combined dataset. The most suitable substitution model was determined using jModelTest v. 2.1.10 software [37,38]. ML analyses were conducted using RAxML-HPC2 on XSEDE on the online CIPRES Portal (https://www.phylo.org/portal2, accessed on 2 January 2023), with a default GTR substitution matrix and 1000 rapid bootstraps. BI analyses were performed using MrBayes v. 3.2.6 [39]. Four Markov chain Monte Carlo (MCMC) chains were run from a random starting tree for 5 million generations, and trees were sampled every 100th generation. The first 25% of the trees were removed as burn-in, and the remaining trees were used to calculate posterior probabilities. A PP value ≥ 0.95 was considered significant. *Fennellomyces linderi* CBS 158.54 was chosen as the outgroup. The newly obtained sequences were deposited in the GenBank database (http://www.ncbi.nlm.nih.gov, accessed on 5 February 2023) under the accession numbers provided in Table 1.

## 3. Results

### 3.1. Phylogenetic Analysis

The ITS and LSU sequences obtained from all isolates were carefully checked with the databases with regards to type of material. A BLAST search of ITS and LSU sequences via the NCBI database indicated that the isolates (CNUFC ISS71, CNUFC S724, and CNUFC CY2204) had highest similarity to *Lichtheimia corymbifera* CBS 429.75 (neotype strain) (GenBank NR_111413; Identities = 91.8%), and *L*. *hyalospora* CBS 173.67 (neotype strain) (GenBank GQ342905; Identities = 94.9%), respectively. A BLAST analysis with ITS and LSU of isolates (CNUFC CY2246 and CNUFC CY2248) showed 99.6% and 100% similarity matches with *L*. *hyalospora* CBS 173.67 (neotype strain) (GenBank NR_111440 and GQ342905), respectively. BLASTn using ITS and LSU regions of CNUFC S871 and CNUFC CY2232 revealed similarities of 95.9% and 99.7% with *L. ornata* CBS 291.66 (type strain) (GenBank NR_111439 and GQ342946), respectively. ITS and LSU sequences of *L*. *ramosa* CNM-CM:CM5398 (GenBank HM104210) and *L*. *ramosa* CBS 582.65 (neotype strain) (GenBank NG_042518) showed 99% and 98.9% homologies with the ITS and LSU sequences of the isolate CNUFC CY2219, respectively.

**Figure 1:**
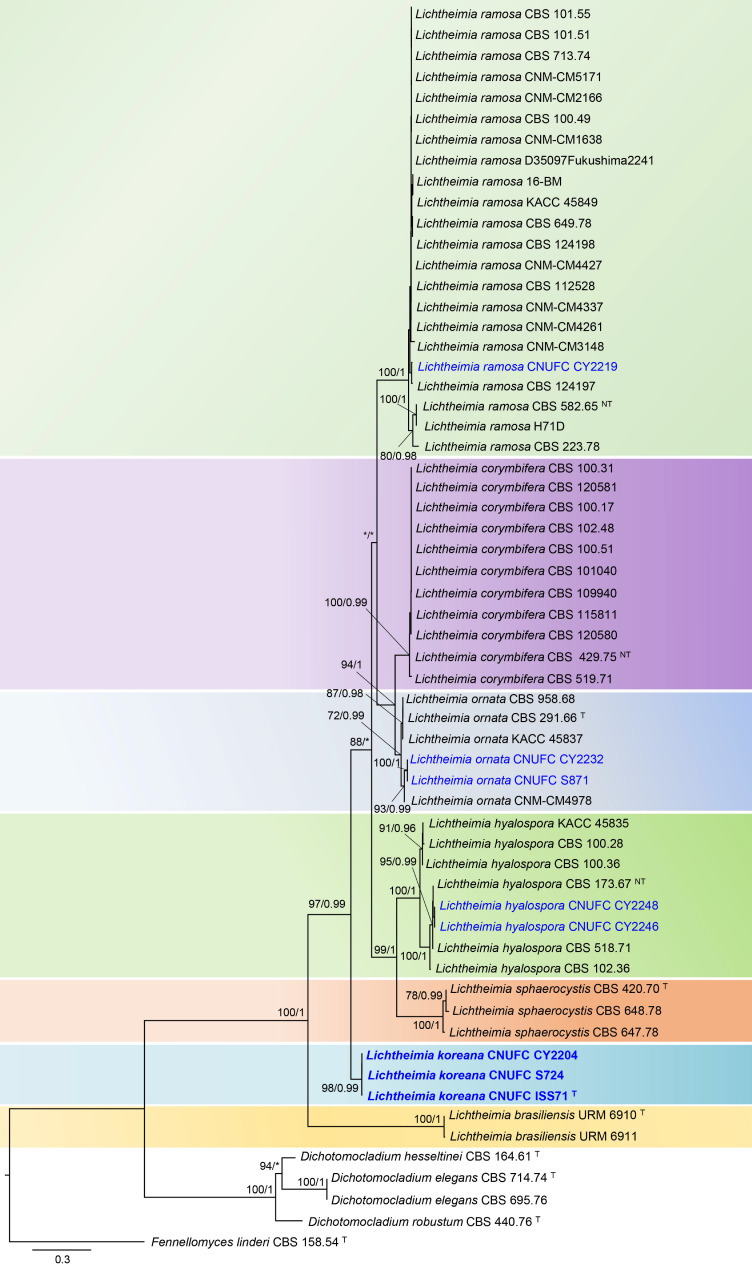
Phylogram generated from the Maximum Likelihood (RA×ML) analysis based on the combined ITS and LSU sequence data of *Lichtheimia* spp. and *Dichotomocladium* spp. The numbers above or below branches represent maximum likelihood bootstrap percentages (**left**) and Bayesian posterior probabilities (**right**). Bootstrap values ≥ 70% and Bayesian posterior probabilities ≥ 0.95 are indicated above or below branches. Bootstrap values lower than 0.95 and 70% are marked with “*”. *Fennellomyces linderi* CBS 158.54 was used as the outgroup. The newly generated sequences are indicated in blue and new species are in bold. ^T^ = type strain; ^NT^ = neotype strain.

The multigene analysis contained 60 taxa, including *Fennellomyces linderi* CBS 158.54 as the outgroup taxon. The concatenated alignment consisted of 1589 characters (including alignment gaps), with 939 and 650 characters used in the ITS and LSU, respectively. The isolates CNUFC ISS71, CNUFC S724, and CNUFC CY2204 formed an independent branch that was well-supported (97% MLBS, 0.99 PP) and clearly distinct from the other *Lichtheimia* species. CNUFC CY2219 clustered with strains of *L. ramosa*, while CNUFC S871 and CNUFC CY2232 clustered with strains of *L*. *ornata*, and CNUFC CY2246 and CNUFC CY2248 clustered with strains of *L*. *hyalospora* (Figure 1).

### 3.2. Taxonomy

**Figure 2:**
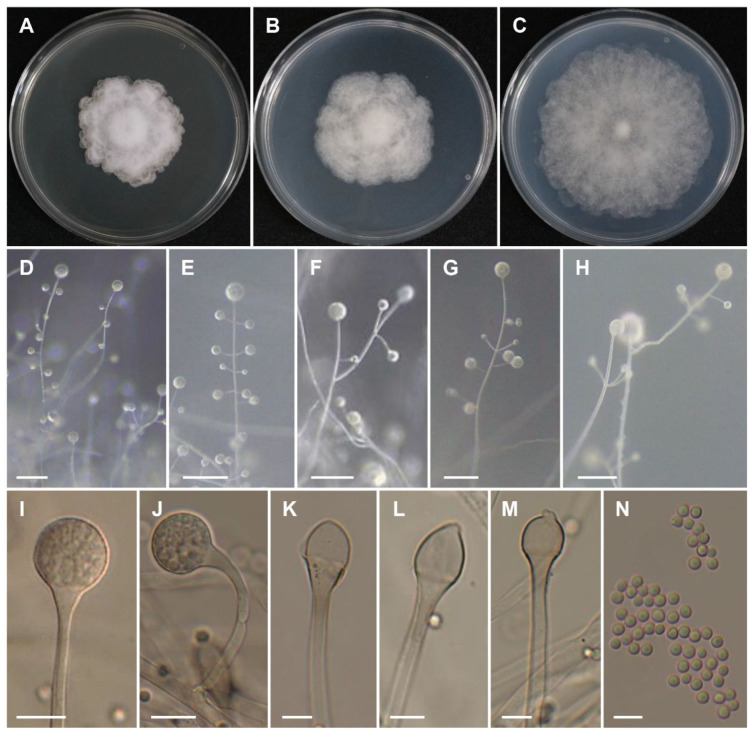
*Lichtheimia koreana* (CNUFC ISS71). (**A**) colony on MEA; (**B**) colony on PDA; (**C**) colony on SMA; (**D**–**H**) branched sporangiophores with sporangia observed under stereomicroscope; (**I**) mature sporangium; (**J**) circinate sporangiophore with sporangium; (**K**–**M**) columellae with or without projection; (**N**) sporangiospores. Scale bars: D–H = 100 μm, I–J = 20 μm, K–N = 10 μm.

*Lichtheimia koreana* Hyang B. Lee, A.L. Santiago & T.T.T. Nguyen, sp. nov. (Figure 2).

Index Fungorum: 900087.

Etymology: Referring to the country from which the species was first isolated.

Description: Colonies on MEA developing slowly, low, white at first, becoming gray with age, reaching a diameter of 37–40 mm after 5 days of incubation at 25 °C; reverse gray and strongly wavy zonate. Sporangiophores hyline to light gray, brown toward the columella in old culture, simple, monopodially or sympodially branched, arising solitarily or up to three at a single place from stolons, 3–8 μm in diameter; branches of sporangiophores hyaline to brown toward columella, erect to slight and strong cirinate, 2.5–4.5 μm wide, and (20–) 35–115 μm long. Terminal sporangia spherical, subpyriform to pyriform, hyaline to gray, slightly yellow to brown in age, 20–38.5 × 19.0–35 μm, smoot-walled; columellae hemispherical, subglobose to oval without projections, hyaline to light brown-gray with age, 12–23.5 × 15–27.5 μm, smooth-walled. Lateral sporangia similar to terminal ones in shape, spherical, subpyriform to pyriform, hyaline to brown, but smaller, 15–27 × 14.5–26.5 μm; columellae smaller, subglobose, oval, tapering, short or long conical, hyaline to light brown-gray with age, 10–16.5 × 8.5–12.5 μm, frequently with one projection at the tip, short, nipple-like, sometimes elongated, or irregular, up to 3 µm long, smooth walled. Collar present or not. Sporangiospores yellow-green, mostly globose, some subglobose, 3.0–4.5 × 3.0–4.0 μm, smooth-walled. Rhizoids branched. Giant cells absent. Chlamydospores not seen. Zygospores not observed. Shape and size of sporangiospores are similar on PDA and MEA, but slightly smaller on SMA (3–5.5 μm in diameter). Sporangia on MEA and SMA (up to 44 μm in diameter) are bigger than those on PDA [(11–) 15–26 µm in diameter].

Habitat: Isolated from *Timomenus komarovi, Theuronema hilgendorfi hilgendorfi, Nephila* sp.

Distribution: Korea.

Specimen examined: REPUBLIC OF KOREA, Kunryang-ri (36°26′16.2″ N 126°46′04.6″ E), Cheongyang-eup, Cheongyang, Chungnam Province, from *Timomenus komarovi*, 24 April 2020, H.B. Lee and J.S. Kim (holotype CNUFC HT2007; ex-type living culture CNUFC ISS71).

Additional material examined: REPUBLIC OF KOREA, in a home garden located on a hill in Kunryang-ri (36°26′16.2″ N 126°46′04.6″ E), Cheongyang-eup, Cheongyang, Chungnam Province, from *Theuronema hilgendorfi hilgendorfi*, 14 June 2020, H.B. Lee (culture CNUFC S724); from *Nephila* sp., 10 Octorber 2022, H.B. Lee (culture CNUFC CY2204).

Media and temperature tests: Colony diameter, 48 h, in mm: SMA 20 °C 14; SMA 25 °C 29; SMA 30 °C 39; SMA 35 °C 36; SMA 40 °C 6; SMA 41 °C no growth; MEA 20 °C 13; MEA 25 °C 18.5; MEA 30 °C 19.5; MEA 35 °C 25; MEA 40 °C 4; MEA 41 °C no growth; PDA 20 °C 10.5; PDA 25 °C 21; PDA 30 °C 23.5; PDA 35 °C 26.5; PDA 40 °C 4; PDA 41 °C no growth. Maximum growth temperature of 40 °C.

**Figure 3:**
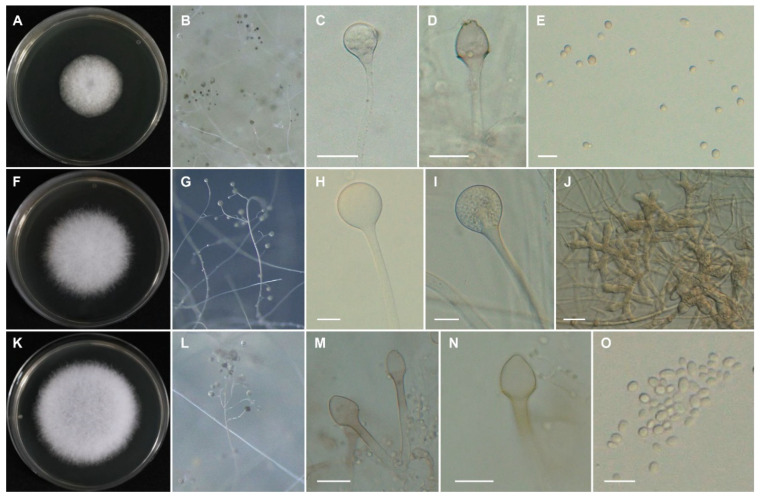
Morphology of *Lichtheimia* spp. *Lichtheimia hyalospora* CNUFC CY2246 (**A**–**E**) [(**A**) colony on MEA at 35 °C. (**B**) sporangiophores with sporangia observed under stereomicroscope. (**C**) sporangium. (**D**) columella with projection. (**E**) sporangiospores]. *Lichtheimia ornata* CNUFC S817 (**F**–**J**) [(**F**) colony on MEA at 35 °C. (**G**) sporangiophores with sporangia observed under stereomicroscope. (**H,I**) young and mature sporangia. (**J**) giant cells formed on PDA]. *Lichtheimia ramosa* CNUFC CY2219 (**K**–**O**) [(**K**) colony on MEA at 35 °C. (**L**) sporangiophores with sporangia observed under stereomicroscope. (**M, N**) columellae with and without collars. (**O**) sporangiospores]. Scale bars: C, D, H, I, M, N = 20 μm, E, O = 10 μm, J = 50 μm.

*Lichtheimia hyalospora* (Saito) Kerst. Hoffman, G. Walther & K. Voigt, Mycological Research 113 (3): 278 (2009); Figure 3A–E.

Basionym. *Tieghemella hyalospora* Saito, Zentralblatt für Bakteriologie und Parasitenkunde, Abteilung 2 17: 103 (1906).

Synonym. *Absidia hyalospora* (Saito) Lendn., Matériaux pour la Flore Cryptogamique Suisse 3 (1): 142 (1908).

       *Mycocladus hyalospora* (Saito) J.H. Mirza (1979).

       *Mycocladus hyalosporus* (Saito) J.H. Mirza, *Mucorales* of Pakistan: 97 (1979).

Descriptions & Illustrations: Hesseltine and Ellis [40] and Alastruey-Izquierdo et al. [7].

Habitat: Isolated from Kurone developed during the manufacture of soy sauce (koji) [7], Fermented food taosi [7], *Manihot esculenta*; stem [7], *Bertholletia excels*; nut [7], soil [41], meju [42], and *Nephila* sp. (this study).

Distribution: Ghana [7], Philippines [7], Japan [7], USA [7], Brazil [41], Korea [42] and this study.

Additional materials examined: REPUBLIC OF KOREA, in a home garden located on a hill in Kunryang-ri (36°26′16.2″ N 126°46′04.6″ E), Cheongyang-eup, Cheongyang, Chungnam Province, from *Nephila* sp., 10 Octorber 2022, H.B. Lee (cultures CNUFC CY2246 and CNUFC CY2248).

*Lichtheimia ornata* (A.K. Sarbhoy) Alastr.-Izq. & G. Walther, Journal of Clinical Microbiology 48 (6): 2164 (2010); Figure 3F–J.

Basionym. *Absidia ornata* A.K. Sarbhoy, Canadian Journal of Botany 43 (8): 999 (1965).

Synonym. *Absidia hesseltinei* B.S. Mehrotra (1967).

       *Absidia hesseltinii* B.S. Mehrotra (1967).

Descriptions & Illustrations: Sarbhoy [43] and Alastruey-Izquierdo et al. [7].

Habitat: Isolated from dung of bird [7], soil [7], *Homo sapiens* (wound) [7], meju [42], soft tissue in nose root [44], *Scolopendra morsitans* and *Theuronema hilgendorfi hilgendorfi* (this study).

Distribution: India [7], Spain [7], China [7,44], and Korea [42] and this study.

Additional materials examined: REPUBLIC OF KOREA, in a home garden located on a hill in Kunryang-ri (36°26′16.2″ N 126°46′04.6″ E), Cheongyang-eup, Cheongyang, Chungnam Province, from *Scolopendra morsitans*, 14 March 2021, H.B. Lee (culture CNUFC S871), from *Theuronema hilgendorfi hilgendorfi* 9 November 2022, H.B. Lee (culture CNUFC CY2232).

*Lichtheimia ramosa* (Zopf) Vuill., Bulletin de la Société Mycologique de France 19: 126 (1903); Figure 3K–O.

Basionym. *Rhizopus ramosus* Zopf, Handbuch der Botanik 4: 587 (1890).

Synonym. *Absidia ramosa* (Zopf) Lendn., Matériaux pour la Flore Cryptogamique Suisse 3 (1): 144 (1908).

       *Mycocladus ramosus* (Zopf) J.H. Mirza, *Mucorales* of Pakistan: 97 (1979).

       *Mucor ramosus* Lindt, Arch. Exp. Path. Pharmacol.: 269 (1886).

       *Absidia corymbifera* var. *ramosa* (Zopf) Coudert, Guide pratique de mycologie médicale: 120 (1955).

       *Mycocladus ramosus* (Zopf) Vánová, Česká Mykologie 45 (1–2): 26 (1991).

       *Mycocladus ramosa* (Zopf) J.H. Mirza (1979).

Descriptions & Illustrations: Ellis and Hesseltine [45].

Habitat: Isolated from soil [7], cow dung [7], guinea-pig lung [7], *Musa sapientum* [7], hay [7], culture contaminant [7], composting soils [22], meju [42], *Homo sapiens* (wound, lung, skin, sputum, gastric juice, pneumonia, bronchoalveolar lavage) [7,46,47,48,49,50], Moutai-flavor Daqu [51], fresh press-mud [52], green coffee bean [53], nuruk [54], *Bos taurus* [55], bovine liver tissue [56], soil [57], bandages [58], ovine milk [59], marine sediments [60], clinical sample [61], and *Theuronema hilgendorfi hilgendorfi* (this study).

Distribution: Indonesia [7], Netherlands [7], Switzerland [7], Ghana [7], India [7,46,49,60], Germany [7,50], Greece [7], Spain [7,59], Belgium [7], Japan [55], China [47,48,51], Mexico [22,57], France [58], Brazil [52,53], Egypt [61], Korea [42,54,56] and this study.

Additional materials examined: REPUBLIC OF KOREA, in a home garden located on a hill in Kunryang-ri (36°26′16.2″ N 126°46′04.6″ E), Cheongyang-eup, Cheongyang, Chungnam Province, from *Theuronema hilgendorfi hilgendorfi* 20 June 2021, H.B. Lee (culture CNUFC CY2219).

### 3.3. Mating Experiments

Zygospores were not produced under any conditions between any of the mating pairs.

### 3.4. Growth Experiments

**Figure 4:**
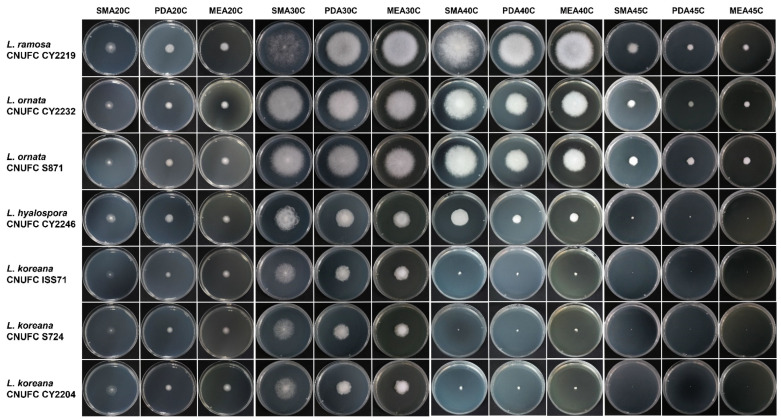
Radial growth determination of *Lichtheimia* species at different temperatures of 20, 30, 40 and 45 °C on SMA, PDA and MEA.

The growth experiments using plates with PDA, MEA, and SMA showed that the choice of media affected the growth of the studied isolates (Figure 4). All isolates grew at temperatures between 20 to 40 °C. Maximum growth was recorded for different species at temperatures ranging from 40 to 47 °C (Table 2). The highest growth rates at all temperatures tested were recorded for *Lichtheimia ramosa* (CNUFC CY2219) and *L*. *ornata* (CNUFC CY2232 and CNUFC S817), respectively. The most favourable growth media for all species was SMA. *Lichtheimia koreana* grew slower on SMA, PDA and MEA than *L*. *hyalospora*, *L*. *ornata* and *L*. *ramosa*. *Lichtheimia hyalospora* (CNUFC CY2246 and CNUFC CY2248) were able to grow at 45 °C, while none of the tested *L*. *koreana* grew at this temperature. Maximum growth temperature for *L*. *koreana* is 40 °C. *Lichtheimia ramosa* (CNUFC CY2219) and *L*. *ornata* (CNUFC CY2232 and CNUFC S817) grew well at 45 °C. However, *L*. *ornata* (CNUFC CY2232 and CNUFC S817) could be distinguished from *Lichtheimia ramosa* (CNUFC CY2219) by its ability to grow at 47 °C, since the maximal growth temperature for *Lichtheimia ramosa* (CNUFC CY2219) was at 46 °C.

## 4. Discussion

The genus *Lichtheimia* contains six accepted species. In this study, *Lichtheimia* isolates obtained from invertebrates in Korea were studied. A new species is described based on evidence from a polyphasic approach.

The data from the combined sequence analysis of two loci (ITS and LSU rDNA) showed that *L*. *koreana* formed well-supported clades (MLBS: 97%, PP: 0.99) (Figure 1). *Lichtheimia koreana* was embedded among the clade of *L*. *brasiliensis* and clade containing *L*. *sphaerocystis* and *L. hyalospora*. *Lichtheimia koreana* shares several similarities with *L*. *brasiliensis*, including optimal growth at 30 to 35 °C, restricted growth at 40 °C, and rhizoid production [8]. However, this species differs from *L*. *brasiliensis* in forming columellae with projections, sporangiophores arising solitarily or up to three at a single place from stolons, and smaller sporangia, while *L*. *brasiliensis* forms columellae with no projections, sporangiophores arising solitary or in pairs from stolon, and sporangia up to 55 μm in diameter [8]. *Lichtheimia sphaerocystis* produces giant cells, whereas this structure is not observed in *L*. *koreana*. *Lichtheimia hyalospora* differs from *L*. *koreana* in its larger sporangia (20–56 μm) and sporangiospores [5.5–9 (–13) μm diameter]) [40]. *Lichtheimia koreana* can also be distinguished from *L*. *corymbifera*, *L*. *ornata*, and *L*. *ramosa* by its maximum growth temperature. The maximum growth temperature for *L*. *corymbifera*, *L*. *ornata*, and *L*. *ramosa* as determined by Alastruey-Izquierdo et al. [7] is 49 °C, 46 °C and 49 °C, respectively, while in our study, *L*. *koreana* exhibited a maximum growth temperature of 40 °C.

The temperature factor for maximum growth is useful to distinguish between species of *Lichtheimia* [7]. For example, at 43 °C, *L*. *ramosa* has higher growth rate than *L*. *corymbifera* and *L*. *ornata*, while *L*. *hyalospora* and *L*. *sphaerocystis* did not grow at this temperature [7]. However, two strains of *L*. *hyalospora* (CNUFC CY2246 and CNUFC CY2248) in this study were able to grow at 43 °C and have a maximum growth temperature of 45 °C. These discrepancies could be attributed to different hosts, seasons of sample collection, and geographical regions. Interestingly, both species, *L*. *ramosa* (CNUFC CY2219) and *L*. *ornata* (CNUFC S871 and CNUFC CY2232) did not grow above 46 and 47 °C, respectively.

All species of *Lichtheimia* grow well at 37 °C, but only three species, namely *L*. *corymbifera*, *L*. *ornata*, and *L*. *ramosa*, have been reported to cause human infections [7,15]. *Lichtheimia koreana* is embedded among clade of *L*. *brasiliensis*, *L*. *sphaerocystis* and *L. hyalospora*, which are not human pathogens [15]. Thus, the pathogenic potential of this new species is probably limited.

*Lichtheimia corymbifera* and *L*. *ramosa*, which represent the most important pathogenic species of *Lichtheimia*, are also isolated from Asian food productions such as meju (soybean based fermented products) and nuruk (a traditional starter culture for brewing alcoholic beverages in Korea) [42,54]. In this study, we isolated *L*. *ramosa* and *L*. *corymbifera* from invertebrates, suggesting that we need to consider the natural environments of these species alongside their ability to infect humans.

Members of *Lichtheimia* are thermotolerant and can grow at a wide range of temperatures from 24 to 50 °C [7]. The ability to grow at high temperatures makes these species valuable in industrial processes. Thus, the potential biological activities of species of *Lichtheimia* obtained from this study should be further examined. It is also necessary to better understand the distribution of these species and their relevance in human and animal diseases.

## 5. Conclusions

A new species, *L*. *koreana,* and three new host records, *L*. *hyalospora*, *L*. *ornata*, *L*. *ramosa,* isolated from invertebrates, were classified based on polyphasic approaches including molecular, morphological, and physiological works. Our findings may contribute to the current knowledge of the species diversity of *Lichtheimia* in Korea. Using poorly studied substrates or hosts for isolation of the fungal species will increase our knowledge of their biodiversity and lead to a better understanding of their specific habitats or niches.

## Figures and Tables

**Table 1:** Taxa, collection numbers, and GenBank accession numbers used in this study.

Species	Strain	Source	Country	GenBank Accession No.	
				ITS	LSU
*Fennellomyces linderi*	CBS 158.54 (T)	Poplin	USA	JN205846	HM849723
*Dichotomocladium elegans*	CBS 714.74 (T)	Soil of a cultivated field	India		JN206555
*Dichotomocladium elegans*	CBS 695.76	Dung of rodent	USA		HM849715
*Dichotomocladium hesseltinei*	CBS 164.61 (T)	Soil of a cultivated field	India		JN206556
*Dichotomocladium robustum*	CBS 440.76	Dung of mouse	USA		JN206557
*Lichtheimia brasiliensis*	URM6910 (T)	Soil	Brazil	KC740486	KC740485
*Lichtheimia brasiliensis*	URM6911	Soil	Brazil	KC740489	KC740484
*Lichtheimia corymbifera*	CBS 429.75 (NT)	Soil	Afghanistan	GQ342878	GQ342903
*Lichtheimia corymbifera*	CBS 100.51	n.a.	n.a	GQ342886	GQ342939
*Lichtheimia corymbifera*	CBS 519.71	n.a.	Japan	GQ342889	GQ342904
*Lichtheimia corymbifera*	CBS 100.17	n.a.	n.a.	GQ342885	GQ342942
*Lichtheimia corymbifera*	CBS 100.31	Aborted cow	n.a.	GQ342879	GQ342914
*Lichtheimia corymbifera*	CBS 102.48	Moldy shoe	India	GQ342888	GQ342910
*Lichtheimia corymbifera*	CBS 101040	Human; keratomycosis	France	GQ342882	GQ342918
*Lichtheimia corymbifera*	CBS 109940	Human; finger tissue	Norway	GQ342881	GQ342917
*Lichtheimia corymbifera*	CBS 115811	Indoor air	Germany	GQ342887	GQ342932
*Lichtheimia corymbifera*	CBS 120580	Human; lung	France	GQ342884	GQ342919
*Lichtheimia corymbifera*	CBS 120581	Human; bronchus	France	GQ342883	GQ342948
*Lichtheimia hyalospora*	CBS 173.67 (NT)	Fermented food taosi	Philippines	GQ342893	GQ342905
*Lichtheimia hyalospora*	CBS 102.36	*Manihot esculenta*; stem	Ghana	GQ342895	GQ342907
*Lichtheimia hyalospora*	CBS 100.28	*Bertholletia excelsa;* nut	USA	GQ342896	GQ342902
*Lichtheimia hyalospora*	CBS 100.36	n.a	n.a	GQ342898	GQ342943
*Lichtheimia hyalospora*	CBS 518.71	Kurone developed during the manufacture of soy sauce (koji)	Japan	GQ342894	GQ342944
*Lichtheimia hyalospora*	KACC 45835	Meju	Korea	JN315003	JN315034
***Lichtheimia hyalospora***	**CNUFC CY2246**	***Nephila* sp.**	**Korea**	**OQ407527**	**OQ383339**
***Lichtheimia hyalospora***	**CNUFC CY2248**	***Nephila* sp.**	**Korea**	**OQ407528**	**OQ383340**
***Lichtheimia koreana* sp. nov.**	**CNUFC ISS71**	***Timomenus komarovi***	**Korea**	**OQ407524**	**OQ383336**
***Lichtheimia koreana* sp. nov.**	**CNUFC S724**	***Theuronema hilgendorfi hilgendorfi***	**Korea**	**OQ407525**	**OQ383337**
***Lichtheimia koreana* sp. nov.**	**CNUFC CY2204**	***Nephila* sp.**	**Korea**	**OQ407526**	**OQ383338**
*Lichtheimia ornata*	CNM-CM4978	Human; wound	Spain	GQ342892	JN206554
*Lichtheimia ornata*	CBS 958.68	n.a	n.a	GQ342890	GQ342936
*Lichtheimia ornata*	CBS 291.66	Dung of bird	India	GQ342891	GQ342946
*Lichtheimia ornata*	KACC 45837	Meju	Korea	JN315004	JN315035
***Lichtheimia ornata***	**CNUFC CY2232**	***Theuronema hilgendorfi hilgendorfi***	**Korea**	**OQ407529**	**OQ383341**
***Lichtheimia ornata***	**CNUFC S871**	***Scolopendra morsitans***	**Korea**	**OQ407530**	**OQ383342**
*Lichtheimia ramosa*	CBS 582.65 (NT)	*Theobroma cacao;* seed	Ghana	GQ342874	GQ342909
*Lichtheimia ramosa*	CBS 223.78	Cocoa soil	n.a	GQ342877	GQ342934
*Lichtheimia ramosa*	CBS 713.74	n.a	n.a	GQ342856	GQ342935
*Lichtheimia ramosa*	CBS 100.49	Cow dung	Indonesia	GQ342858	GQ342940
*Lichtheimia ramosa*	CBS 101.51	Guinea pig; lung	Netherlands	GQ342859	GQ342945
*Lichtheimia ramosa*	CBS 101.55	Human; cornea	Switzerland	GQ342865	GQ342947
*Lichtheimia ramosa*	CBS 649.78	Cultivated field soil	India	GQ342849	GQ342912
*Lichtheimia ramosa*	CBS 112528	Human, wound; double infection with Candida albicans	Germany	GQ342850	GQ342913
*Lichtheimia ramosa*	CBS 124197	Human	Greece	GQ342870	GQ342951
*Lichtheimia ramosa*	CBS 124198	Culture contaminant	Netherlands	GQ342848	GQ342906
*Lichtheimia ramosa*	CNM-CM1638	Human, gastric juice	Spain	GQ342866	GQ342954
*Lichtheimia ramosa*	CNM-CM2166	Human; sputum	Spain	GQ342863	GQ342926
*Lichtheimia ramosa*	CNM-CM3148	Human; corneal exudate	Spain	GQ342872	GQ342925
*Lichtheimia ramosa*	CNM-CM4427	Human; bronchoaspirate	Spain	GQ342853	GQ342931
*Lichtheimia ramosa*	CNM-CM4337	Human; skin	Spain	GQ342852	GQ342920
*Lichtheimia ramosa*	CNM-CM4261	Human; lung	Spain	GQ342854	GQ342953
*Lichtheimia ramosa*	CNM-CM5171	Human	Belgium	GQ342864	GQ342927
*Lichtheimia ramosa*	H71D	Soil	Mexico	KY311837	-
*Lichtheimia ramosa*	D35097Fukushima2241	*Bos taurus*	Japan	LC643024	
*Lichtheimia ramosa*	16-BM	Bandages	France	KX764883	MG772622
*Lichtheimia ramosa*	KACC 45849	Meju	Korea	JN315006	JN315037
***Lichtheimia ramosa***	**CNUFC CY2219**	***Theuronema hilgendorfi hilgendorfi***	**Korea**	**OQ407531**	**OQ383343**
*Lichtheimia sphaerocystis*	CBS 647.78	Dung of mouse	India	GQ342899	GQ342911
*Lichtheimia sphaerocystis*	CBS 420.70 (T)	n.a	India	GQ342900	GQ342933
*Lichtheimia sphaerocystis*	CBS 648.78	Soil	India	GQ342901	GQ342916

Isolates and accession numbers determined in the current study are indicated in bold. CBS: Centraalbureau voor Schimmelcultures, Utrecht, The Netherlands; CNM-CM: Instituto de Salud Carlos III National Centre of Microbiology, Madrid, Spain; CNUFC: Chonnam National University Fungal Collection, Gwangju, Korea; KACC: Korean Agricultural Culture Collection; URM: Micoteca URM, Universidade Federal de Pernambuco, Recife, Brazil. Type and neotype strains are denoted by T and NT, respectively. n.a: not available.

**Table 2:** Species tested and maximum temperature growth on MEA, PDA and SMA.

Species	Strain	Maximum Growth Temperature (°C)	Temperature without Growth (°C)
*Lichtheimia koreana* sp. nov.	CNUFC ISS71	40	41
*Lichtheimia koreana* sp. nov.	CNUFC S724	40	41
*Lichtheimia koreana* sp. nov.	CNUFC CY2204	40	41
*Lichtheimia hyalospora*	CNUFC CY2246	45	46
*Lichtheimia hyalospora*	CNUFC CY2248	45	46
*Lichtheimia ornata*	CNUFC CY2232	47	48
*Lichtheimia ornata*	CNUFC S871	47	48
*Lichtheimia ramosa*	CNUFC CY2219	46	47

## Data Availability

All sequences generated in this study were submitted to GenBank.
